# Body mass index as a dominant risk factor for metabolic syndrome among indonesian adults: a 6-year prospective cohort study of non-communicable diseases

**DOI:** 10.1186/s40795-024-00856-8

**Published:** 2024-03-04

**Authors:** Nurul Dina Rahmawati, Helen Andriani, Fadila Wirawan, Latifah Farsia, Alexander Waits, Khobir Abdul Karim Taufiqurahman

**Affiliations:** 1 Department of Public Health Nutrition, Faculty of Public Health, Universitas Indonesia, 16424 Depok, West Java Indonesia; 2 Department of Health Policy and Administration, Faculty of Public Health, Universitas Indonesia, 16424 Depok, West Java Indonesia; 3 Institute of Public Health, School of Medicine, National Yang-Ming Chiao Tung University, Taipei, 112 Taiwan; 4 Department of Biostatistics, Faculty of Public Health, Universitas Indonesia, 16424 Depok, West Java Indonesia

**Keywords:** Metabolic syndrome (MetS), Body mass index (BMI), Risk factors, Prospective cohort study, Non-communicable diseases, Indonesian adults

## Abstract

**Background:**

Non-communicable diseases (NCDs), notably cardiovascular disease and type 2 diabetes mellitus, are largely driven by metabolic syndrome (MetS), a cluster of critical risk factors. Despite extensive research, the progression of MetS, especially in Indonesia, has received limited attention. This research tracks adult MetS risk dynamics in a populous Bogor District cohort, providing crucial insights into its evolving nature.

**Methods:**

This prospective open cohort study analysed secondary data from the Special Research - Cohort Study of Non-Communicable Diseases by the Ministry of Health, Republic of Indonesia from 2011 to 2018. The final sample was 1,376 Indonesian adult participants, all residents of Bogor District. MetS outcome, dietary assessment, physical activity, and biomarkers were analysed every two consecutive years.

**Results:**

The risk of overweight and obese participants developing MetS was 2.4 and 4.4 times higher, respectively (95% CI: 1.176–3.320 and 3.345–5.740) than those with body mass index (BMI) in the normal range. Participants who reported less intentional physical exercise had a MetS risk 1.5 times higher (95% CI: 1.034–2.109) than those with more intentional physical exercise. The role of diet is also significant, evidenced by a 30% reduction in MetS risk for people with fat intakes in the 2nd quartile compared to the 1st quartile (95% CI: 0.505–0.972). Meanwhile, a carbohydrate intake in the 2nd quartile increased the risk of MetS 1.5 times (95% CI: 1.063–2.241) in comparison with the 1st quartile.

**Conclusions:**

Notably, participants with underweight BMI exhibited the highest cumulative survival of MetS, while those with obese BMI recorded the lowest cumulative survival. There is an urgent need for strategic interventions to enhance the existing early detection and NCD monitoring program. This involves a targeted focus on promoting a community-based healthy lifestyle in the Bogor District. The study emphasizes the importance of tailored public health measures to address specific risk factors identified in the local context, aiming to mitigate the prevalence and impact of MetS in the population.

**Supplementary Information:**

The online version contains supplementary material available at 10.1186/s40795-024-00856-8.

## Background

The third of the UN’s Sustainable Development Goals (SDGs) is to “Ensure healthy lives and promote well-being for all at all ages”. Encompassed within this is Target 3.4 which aims to reduce premature mortality from non-communicable diseases (NCDs) by one-third by 2030 through prevention and treatment [1]. Despite the implementation of public health policies around the world to address the issue, the prevalence and burden of NCD including diabetes mellitus, obesity and stroke, continues to rise [2, 3]. Forty-one million deaths per year (equivalent to 71% of all deaths globally) are attributed to NCDs, of which 85% are premature deaths that occur in low- and middle-income countries, including Indonesia [4].

Although NCDs that occur singularly represent a significant public health challenge, they often occur in combination, increasing the burden on healthcare services. Metabolic syndrome (MetS) refers to a group of risk factors that collectively increase the risk of cardiovascular disease, insulin resistance and diabetes mellitus, and vascular and neurological complications such as cerebrovascular diseases [5–7]. Metabolic disorders are categorised as MetS if an individual has at least three of the following conditions: (1) abdominal obesity, (2) high triglyceride levels, (3) low levels of high-density lipoprotein cholesterol (HDL), (4) elevated fasting glucose levels, (5) hypertension [8, 9].

Studies suggest that populations with an imbalanced eating pattern [10–12], individuals with low levels of physical inactivity [13, 14], certain racial and ethnic groups [9], those with a family history of diabetes (sibling or parent with diabetes), women with a history of polycystic ovarian syndrome, and older adults [15] are more likely to suffer from MetS [9].

In Indonesia, the high prevalence of MetS is also an alarming issue. The prevalence is significantly higher compared to other countries (39%, 29.2%, and 30% in Indonesia, Dutch, and Indian adults, respectively) [6, 16], ranging from 0 to 50% of provincial and ethnic prevalence [5] that may be partially attributed to the susceptibility of Asian populations to metabolic disorders [17]. Without proper management and prevention, MetS can exacerbate the national burden of diseases and healthcare costs, which is particularly relevant to Indonesia following implementation of the National Health Insurance scheme (JKN). JKN aims to provide universal health coverage to the entire population from the shock of sudden health crises, including NCDs, which are the main burden in health financing in the JKN program [18]. In 2021, the amount of JKN claim costs for the NCD was around IDR 18 trillion or USD 1.2 billion (consuming 37% of the total costs) which increased to IDR 24 Trillion or USD 1.5 Billion in 2022. NCDs not only exhaust the resources for provision of healthcare services due to their direct cost but are additionally associated with significant indirect costs to society such as productivity losses [19–22]. By 2024, the Indonesian government will target all 514 districts/cities to detect NCD risk factors early. In addition, since 2014, the government also has a chronic disease management program (Prolanis) to improve the quality of life of JKN participants and ensure the effectiveness and efficiency of the health care cost.

In order to develop effective prevention programs, several cohort studies have been conducted to better understand the risk factors for MetS and examine MetS components [23–25], yet this prior research has limitations including relatively short follow-up times (one to four years) [26], variation in diagnostic criteria for MetS, or studies on populations of different ethnicities, which may affect generalisability to the Indonesian context [27, 28]. Another study also explored MetS incidence using a similar six-year cohort, but did not rigorously select participants based on complete MetS component profiles and medication usage, which could introduce bias [29]. Due to these limitations, there is still a requirement to understand the dynamics of MetS among the Indonesian population and gain a better understanding of the aspects of prevention and promotion. This is highly relevant for Indonesia given that healthcare costs are borne by the government following the implementation of JKN.

To enhance the quality of evidence and provide a more comprehensive understanding of Metabolic Syndrome (MetS) risk factors, this study utilizes data from the Non-Communicable Disease Cohort, conducted between 2011 and 2018 in the Bogor District Residence of West Java, Indonesia. This dataset, characterized by lesser ethnic diversity and the use of uniform diagnostic criteria, along with consistent medical testing methods, offers a unique platform for our investigation. Our research notably extends existing efforts by conducting a longitudinal examination of MetS cumulative survival rates across a broader segment of the adult population over this extended period. This approach not only deepens our understanding of MetS progression in Indonesia but also highlights the influence of diverse lifestyle and environmental factors within this specific demographic context. By focusing on these aspects, our study provides critical insights for the early detection and management of MetS, thus underlining the novelty and significance of our work in the broader landscape of MetS research. This study aims to understand the changes in MetS risk among Indonesian adults with regard to specific risk factors. The results of this study could provide evidence for regional and national health policymakers to design the most appropriate intervention strategy.

## Method

### Data collection and participants

This study analysed secondary data from the Special Research - Cohort Study of Non-Communicable Diseases (NCD) by the Ministry of Health, Republic of Indonesia. This population-based prospective cohort study began in 2011 and involved around 5,000 participants from 3,441 households reside in five out of eleven villages in Bogor Tengah Sub-district, Bogor City, West Java Provinces, Indonesia. The study employed total population sampling of participants aged 25 years and older who were willing to have routine medical checkups. Additional participants could be recruited at any point during the study (open cohort). Data collectors were local healthcare professionals in collaboration with the Primary Health Care Centre (Puskesmas) and community health cadres, under the supervision of the Health Research Board, Ministry of Health. Participants were monitored three times per year and had a comprehensive medical checkup every two years. This study employed three phases of participant recruitment with initial screenings or examinations conducted in 2011 (phase 1), 2012 (phase 2), and 2015 (phase 3), resulting in the addition of 394 participants to the existing cohort. Despite efforts to maintain participant engagement, we observed a 9% dropout rate over the 6-year observation period, primarily due to death, illness, relocation, and work commitments preventing attendance at scheduled monitoring and examinations [30]. As an open cohort population-based prospective study, it is considered a dynamic cohort that can identify the incidence of diseases [30, 31].

**Fig. 1:**
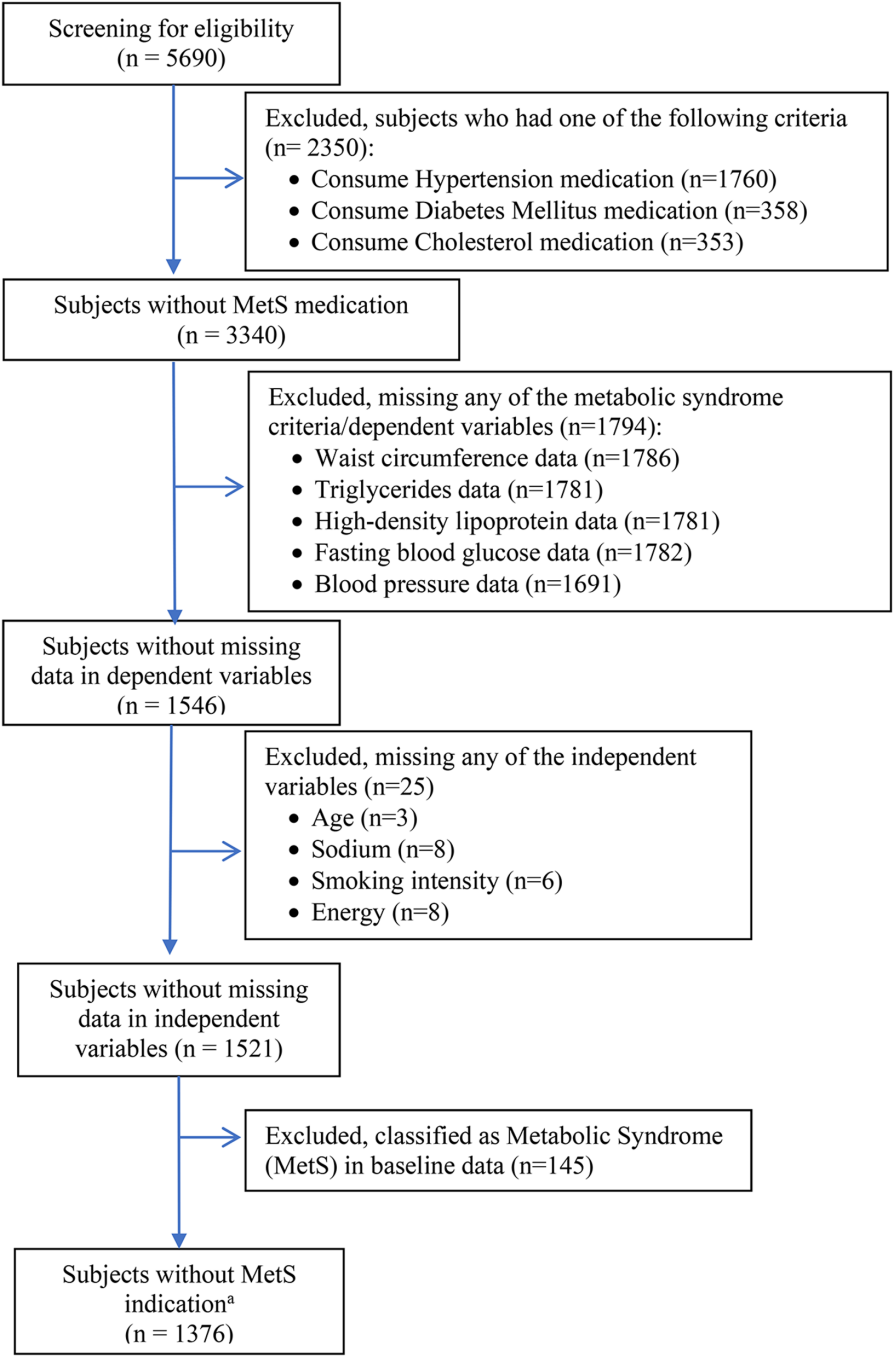
Flow chart of inclusion and exclusion criteria. ^a^ The screening process pertains exclusively to the baseline assessment only

In this study, the initial number of participants was 5,690 adults aged 25 years and above as provided in the dataset. The eligibility criteria were to have complete biomarkers data from baseline (2011) to endline (2018) with no history of MetS or medication regimen related to MetS risk factors (e.g. antihypertensives). We also excluded participants with incomplete independent variables data, including smoking history. The selection process left 1,376 participants to be included in this study (Fig. 1). The meticulous selection was intended to ensure robust findings and distinguish this study form others utilizing the same database.

### Study variables

The observed outcome, MetS, was defined using criteria modified from the National Cholesterol Education Program’s Adult Treatment Panel (NCEP ATP) III criteria, considered for its widely recognized, clinically practical, flexibility and relevance in our study compared to other criteria, namely International Diabetes Foundation (IDF) [32, 33], which states an individual must experience at least three of five conditions as follows: 1) abdominal obesity (waist circumference ≥ 90 cm for men or ≥ 80 cm for women; 2) triglycerides ≥ 150 mg/dL; 3) high-density lipoprotein cholesterol (HDL) < 40 mg/dL for men or < 50 mg/dL for women; 4) fasting blood glucose ≥ 100 mg/dL; and 5) hypertension (systolic blood pressure ≥ 130 mmHg and/or diastolic ≥ 85 mmHg) [33].

Anthropometric measurements, clinical measurements, dietary assessment, sociodemographic profiles, and other risk factors were measured and observed by trained operators and standardised tools. Anthropometric measurements include weight, height, and abdominal circumference. Body mass index (BMI) was calculated from weight and height, then classified based on Asia-Pacific BMI as follows: underweight (< 18.50 kg/m^2^); normal (18.50–22.99 kg/m^2^); overweight (23.00–24.99 kg/m^2^); and obese (≥ 25.0 kg/m^2^) [34]. Clinical measurements included blood glucose and blood lipid profiles (LDL, HDL, triglyceride, total cholesterol).

Food intake was recorded using a single 24-hour dietary recall. It was conducted for each participant in the early stage of the study and during the monitoring (once every year) or 7 times in total. The dietary information was then assessed for energy, carbohydrate, protein, fat, and sodium composition using Nutrisurvey™. The values of energy, carbohydrate, protein, fat, and sodium intake per day were classified into quartiles, with the lowest quartile (Quartile-1) representing the 25% of participants with the lowest consumption in the study population.

Sociodemographic profiles include education, occupation, and the availability of health insurance. Other identified risk factors include smoking, stress, and physical activity. Physical activity was assessed using standardized questionnaires, that is International Physical Activity Questionnaire (IPAQ) developed by WHO to assess physical activity. It covers several components, such as intensity, duration, and frequency, and it assesses three domains in which physical activity is performed (occupational physical activity, transport-related physical activity, and physical activity during discretionary or leisure time). It was then categorised based on the number of minutes spent on low, moderate, and vigorous-intensity activities daily, self-reported by participants. The number of minutes was converted to metabolic equivalent of task (MET) where: (1) walking MET-minutes/week = 3.3 x walking minutes x walking days, (2) moderate MET-minutes/week = 4.0 x moderate-intensity activity minutes x moderate days, and (3) vigorous MET-minutes/week = 8.0 x vigorous-intensity activity minutes x vigorous-intensity days. The result was subsequently further classified into sufficient physical activity levels (≥ 600 MET-minutes per week) and insufficient physical activity levels (< 600 MET-minutes per week) [35]. A sub-variable for intentional physical exercise was also based on the self-reported number of minutes spent on moderate and vigorous intensity physical exercise and recreational sport. The number of minutes was then classified based on the WHO recommendation for physical activity into sufficient (at least 75 min of vigorous or 150 min of moderate activities per week) and insufficient (less than 75 min of vigorous or 150 min of moderate activities per week) [36]. To adjust for variation in exercise intensity, the reporting of vigorous activity was counted as twice that of the moderate activities, and the cut-off for the grouping was set at 150 min of activities. We did the stratification because the type of the occupation in this population was dominated by manual labour (blue collar worker) which may cause bias in the physical activity estimation, as it does not necessarily mean that they have intentional physical exercise. MetS outcome, dietary assessment, and biomarkers were analysed every two consecutive years based on the availability of the data. Several measurements were recorded, including at baseline in two phases: the year 2011 and 2012, and three follow-up measurements in 2013/2014 (2nd year), 2015/2016 (4th year), and 2017/2018 (6th year) were chosen based on the availability of comprehensive data. Meanwhile, the remaining variables: age, physical activity, smoking status, smoking intensity, stress, education level, occupation, health insurance, family history of diabetes mellitus and heart attack (mortality and morbidity experienced by participants’ parents and grandparents), pregnancy and menopausal status, and delivery of a macrosomic new-born (≥ 4,000gr) were measured once at baseline.

### Data analysis

Descriptive statistics were used to examine all variables. Mean and standard deviation (SD) were used to describe numerical values. We employed categorical data analysis, supplemented by non-parametric tests (McNemar, Wilcoxon Mean-Rank, and Friedman) for skewed numerical data. This approach ensures robustness in our bivariate analysis, aligning with the nature of our data and enhancing the validity of our findings. Association between outcome variables and potential risk factors are estimated using Cox Regression with a 5% significance level. We used a person-time of observation to represent the duration between the baseline and MetS occurrence (year-to-MetS). This study’s outcome was to obtain the adjusted Hazard Ratios (HR) and 95% confidence intervals (95% CIs) for all predictors retained in the final model. Statistical analysis of the data was performed using Stata 17 and SPSS Software version 26.

## Results

**Fig. 2:**
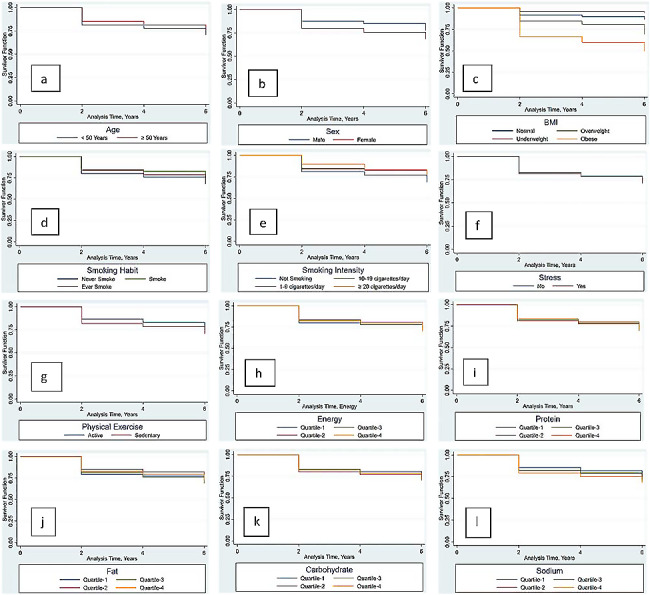
Overall survival rates based on age (a), Sex (b), BMI (c), Smoking Habit (d), Smoking Intensity (e), Stress (f), Physical Exercise (g), Energy Intake (h), Protein Intake (i), Fat Intake (j), Carbohydrate Intake (k), Sodium Intake (l) in years

In this study, 1,521 participants were initially observed; 145 were excluded for meeting MetS criteria, leaving 1,376 for a six-year analysis. Within this period, 28.6% developed MetS. Most participants had a normal BMI (39.6%), while 18.7% and 33.3% were overweight and obese. The majority of participants were females (66.3%), under 50 years (78.5%), lacked health insurance (62.72%), worked as government officers (35.25%), completed secondary school (61.92%), current/ex-smokers (50.9%), experienced no stress (58.0%), and showed insufficient intention to exercise (88.5%). Participant characteristics are detailed in Table 1. In our study, the median survival time for obese individuals was observed to be 6 years, with an incidence rate of MetS at 47.72 per 1000 person-years over a 6-year observation period.By the second year, 17.7% of participants developed MetS, increasing to 21.2% in the fourth year and 28.6% by the sixth year (*p* < 0.001). Notably, waist circumference, triglycerides, and fasting blood glucose levels significantly increased over time (*p* < 0.001), while low HDL and hypertension showed fluctuations. Obesity prevalence rose from 33.3 to 44.9%, with corresponding decreases in normal and underweight BMI categories. In the second year, obesity was most prevalent (38.2%), with a decline in normal BMI (36.0%). Dietary intakes of energy, protein, fat, and sodium escalated, except for carbohydrates, as detailed in Table 2. However, energy, protein, and sodium intakes in year 2 did not significantly differ from baseline. In our analysis, presented in Fig. 2**(a-l)**, several variables affected the survival rate of Metabolic Syndrome (MetS) by the end of the six-year study. Overweight, obesity, sedentary lifestyles, and high carbohydrate consumption were linked to an increased MetS risk compared to normal BMI, intention to exercise, and low carbohydrate intake. Notably, participants with underweight BMI exhibited the highest cumulative survival rate at 94% in the sixth year. Conversely, those with obese BMI showed the lowest rates, from 66.4% at baseline to 49.3% at endline. Age played a significant role, with younger participants (< 50 years) having lower survival rates than older ones ( > = 50 years). Gender differences were also evident; females had a lower cumulative survival rate (68.3%) compared to males (77.4%).

**Table 1:** Participants’ characteristics from baseline to endline (*N* = 1,376)

Characteristics	n (%)
MetS^a^	
No	982 (71.4)
Yes	394 (28.6)
Body Mass Index (BMI)^b^	
Underweight	116 (8.4)
Normal	545 (39.6)
Overweight	257 (18.7)
Obese	458 (33.3)
Age^b^	
< 50	1080 (78.5)
≥ 50	296 (21.5)
Sex^b^	
Male	464 (33.7)
Female	912 (66.3)
Health Insurance Ownership^b^	
Public	479 (34.81)
Private	14 (1.02)
Public and Private	20 (1.45)
None	863 (62.72)
Occupation^b^	
Civil servant	485 (35.25)
Housemaid	437 (31.76)
College Students	31 (2.25)
Self-employed	110 (7.99)
Private sector	11 (0.8)
Labor	54 (3.92)
Others	16 (1.16)
Not working	232 (16.86)
Educational Background^b^	
No formal education	11 (0.8)
Did not complete primary school	125 (9.08)
Primary school	313 (22.75)
Secondary school	852 (61.92)
University	75 (5.45)
Smoking habit ^b^	
Never smoked	676 (49.1)
Ex-smoker	227 (16.5)
Smoke	473 (34.4)
Smoking intensity ^b^	
Not smoking	960 (69.8)
1–9 cigarette(s) per day	226 (16.4)
10–19 cigarettes per day	160 (11.6)
≥ 20 cigarettes per day	30 (2.18)
Stress ^b^	
No	798 (58.0)
Yes	578 (42.0)
Physical activity ^b^	
Overall physical activity	
Sufficient	1246 (90.5)
Insufficient	130 (9.5)
Intention to physical exercise	
Sufficient	158 (11.5)
Insufficient	1218 (88.5)

^a^ Presented from baseline to endline

^b^ Presented at baseline

**Table 2:** Distribution of components of metabolic syndrome and dietary intake based on cohort periods

Characteristics	n (%) *N* = 1,376			Cohort Periods (in year)			
	0 (2011/2012)	p-value (year 2 vs. 0)	2 (2013/2014)	p-value (year 4 vs. 2)	4 (2015/2016)	p-value (year 6 vs. 4)	6 (2017/2018)
Metabolic Syndrome Prevalence							
Yes	0 (0)	< 0.001*	244 (17.7)	< 0.001*	292 (21.2)	< 0.001*	394 (28.6)
No	1,376 (100)		1,132 (82.3)		1,084 (78.8)		982 (71.4)
Waist Circumference (cm)	76.6 ± 10.0 (50–115)	< 0.001**	79. 8 ± 10.1 (49–120)	< 0.001**	82.3 ± 9.8 (56–121)	< 0.001**	83.6 ± 10.0 (54–127)
Abdominal Obesity							
Yes	396 (28.8)	< 0.001*	509 (37.0)	< 0.001*	659 (47.9)	< 0.001*	709 (51.5)
No	980 (71.2)		867 (63.0)		717 (52.1)		667 (48.5)
Triglycerides Level (mg/dL)^c^	92.9 ± 45.6 (30–787)	< 0.001**	101.8 ± 63.3 (30–1305)	0.544**	102.0 ± 57.2 (32–1114)	< 0.001**	112.9 ± 65.59 (32.45–894)
Normal	1,270 (92.3)	< 0.001*	1,220 (88.7)	0.243*	1,205 (87.6)	< 0.001*	1,125 (81.8)
High	106 (7.7)		156 (11.3)		171 (12.4)		251 (18.2)
HDL Level (mg/dL)^c^	51.5 ± 10.8 (24–98)	0.446**	51.6 ± 11.7 (26–173)	< 0.001**	53.0 ± 10.9 (22–96)	< 0.001**	50.6 ± 11.1 (26–101.5)
Normal	961 (69.8)	0.085*	931 (67.7)	< 0.001*	1,025 (74.5)	< 0.001*	876 (63.7)
Low	415 (30.2)		445 (32.3)		351 (25.5)		500 (36.3)
Fasting Blood Glucose Level (mg/dL)^c^	83.3 ± 9.2 (56–134)	< 0.001**	86.3 ± 10.6 (58–151)	< 0.001**	87.6 ± 9.3 (63–202)	< 0.001**	97.6 ± 18.4 (59–349)
Normal	1,371 (99.6)	0.763*	1,370 (99.6)	0.782*	1,369 (99.5)	< 0.001*	1,311 (95.3)
Diabetes Mellitus	5 (0.4)		6 (0.4)		7 (0.5)		65 (4.7)
Systolic Blood Pressure (mmHg)^c^	115.2 ± 12.1 (81–163)	0.143**	115.7 ± 12.4 (86–188)	0.009**	116.4 ± 12.7 (84–185)	< 0.001**	120.5 ± 13.8 (83–186)
Diastolic Blood Pressure (mmHg)^c^	74.1 ± 8.5 (45–103)	< 0.001**	92.5 ± 10.2 (64–142)	< 0.001**	75.2 ± 8.6 (53–103)	< 0.001**	79.2 ± 9.1 (51–121)
Hypertension^d^							
Yes	211 (15.3)	< 0.001*	1,075 (78.1)	< 0.001*	280 (20.3)	< 0.001*	456 (33.1)
No	1,165 (84.7)		301 (21.9)		1,096 (79.7)		920 (66.9)
Body Mass Index (BMI)							
Underweight (< 18.5 kg/m^2^)	116 (8.4)	< 0.001***	104 (7.6)	0.001***	100 (7.3)	< 0.001***	86 (6.3)
Normal (18.5–22.9 kg/m^2^)	545 (39.6)		496 (36.0)		460 (33.4)		423 (30.7)
Overweight (23–24.9 kg/m^2^)	257 (18.7)		251 (18.2)		265 (19.3)		249 (18.1)
Obese (≥ 25.0 kg/m^2^)	458 (33.3)		525 (38.2)		551 (40.0)		618 (44.9)
Dietary Intake^c^							
Energy (kcal)	1721.8 ± 646.7 (185.6–6352.5)	0.992**	1717.3 ± 629.8 (248.5–4838.0)	< 0.001**	1891.8 ± 694.7 (36.0–5537.7)	< 0.001**	2036.3 ± 784.8 (248.8–5733.1)
Carbohydrate (gr)	259.0 ± 102.5 (40.7–1097.3)	0.001**	247.0 ± 94.9 (36.6–839.4)	< 0.001**	263.7 ± 102.0 (7.45–814.4)	< 0.001**	154.8 ± 133.2 (10.22–619.2)
Protein (gr)	57.0 ± 23.2 (3.0–186.3)	0.091**	55.6 ± 23.0 (4.1–265.8)	< 0.001**	61.7 ± 27.8 (0.8–417.7)	< 0.001**	180.9 ± 129.5 (15.3–788.1)
Fat (gr)	52.4 ± 27.3 (0.9–182.9)	< 0.001**	58.4 ± 33.1 (1.5–400.0)	< 0.001**	70.3 ± 34.8 (0.23–295.0)	< 0.001**	80.5 ± 38.7 (7.7–278.76)
Sodium (mg)	1652.8 ± 1605.6 (13.9–15135.5)	0.153**	1508.5 ± 1021.4 (66.5–8264.5)	< 0.001**	1862.2 ± 2851.0 (1.1–77877.3)	< 0.001**	2306.8 ± 1317.1 (173.6–8473.2)

*McNemar Test

**Wilcoxon mean-rank Test

***Friedman Test

^c^ Presented in mean ± SD (min-max)

^d^ Based on systolic and diastolic blood pressure measurement, not based on clinical diagnosis of hypertension

Smoking habits impacted survival, where heavy smokers (> 20 cigarettes per day) had lower survival rates at endline (76.7%) than lighter smokers. Surprisingly, ex-smokers and non-smokers also showed lower survival (70.5% and 67.8%, respectively) compared to those who smoked moderately (77.0%). Participants reporting stress had slightly lower survival rates (70.9%) than non-stressed ones (71.7%). Physical activity levels were critical. Participants with insufficient physical activity had higher cumulative survival at baseline (80.7%) compared to those sufficiently active (70.4%). However, when considering intentional exercise (like sports or recreational activities), those engaging in such exercises had about 8% higher survival than those who did not.

Dietary intake also influenced survival rates. While most nutrients showed fluctuating trends, those in the highest quartile of energy consumption had the lowest survival (69.2%). A similar trend was observed with sodium intake, where higher consumption correlated with lower survival rates, declining from 74.7% in the lowest quartile to 68.0% in the highest.

Multivariate analysis showed that factors influencing MetS survival were BMI, intended exercise and fat and carbohydrate intakes. Participants with underweight BMI had a lower risk of developing MetS (HR = 0.4, 95% CI 0.194–0.919) that indicates a 60% lower risk of developing Metabolic Syndrome (MetS) compared to those with a normal BMI, while overweight and obese participants were 2.4 and 4.4 times more likely to develop MetS (95% CI 1.176–3.320 and 3.345–5.740, respectively) compared to those with normal BMI. In addition, participants who reported less intention to exercise had a MetS risk 1.5 times higher (95% CI 1.034–2.109) than those who were physically active. Dietary fat intakes are also important, evident by an around 30% reduction in MetS risk for fat consumption in the 2nd quartile compared to the 1st quartile On the other hand, carbohydrate intakes in the 2nd quartile increased the risk of MetS 1.5 times (95% CI 1.063–2.241) in comparison to the 1st quartile, and the risk tended to increase in line with higher consumption. Age, sex, smoking habit and intensity, stress, and energy, protein and sodium intake were considered to be confounders (Table 3).

**Table 3:** The model of the cox regression for the 6-year survival rate of MetS

Risk Factors	HR	95% CI	
Age (< 50 Years Old)			
>= 50 Years Old	0.908	0.703–1.174	
Sex (Male)			
Female	0.940	0.680–1.302	
BMI (Normal)^a^			
Underweight	0.422	0.194–0.919	
Overweight	2.415	1.176–3.320	
Obese	4.381	3.345–5.740	
Smoking (No)			
Ever	0.972	0.727–1.300	
Yes	0.938	0.622–1.415	
Smoking Intensity (Not at all)			
1–9 cigarettes/day	1.007	0.642–1.580	
10–19 cigarettes/day	0.996	0.597–1.661	
>= 20 cigarettes/day	0.826	0.370–1.846	
Stress (No)			
Yes	1.053	0.859–1.292	
Intended Physical Exercise (Active)^b^			
Light/Sedentary	1.476	1.034–2.109	
Energy (Q-1)			
Q-2	0.649	0.408–1.033	
Q-3	0.589	0.307–1.131	
Q-4	0.610	0.256–1.453	
Protein (Q-1)			
Q-2	1.179	0.824–1.686	
Q-3	1.201	0.783–1.845	
Q-4	1.210	0.729–2.009	
Fat (Q-1)^c^			
Q-2	0.701	0.505–0.972	
Q-3	0.943	0.643–2.570	
Q-4	0.836	0.524–1.333	
Carbohydrate (Q-1)^d^			
Q-2	1.543	1.063–2.241	
Q-3	1.560	0.947–2.569	
Q-4	1.735	0.933–3.229	
Sodium (Q-1)			
Q-2	1.082	0.806–1.453	
Q-3	1.161	0.868–1.553	
Q-4	1.196	0.884–1.618	

HR = Hazard Ratio; * significance *p* < 0.05; ** *p* < 0.01

^a^Adjusted for age, sex, smoking status, smoking intensity, stress, intended physical exercise, and all the food intakes

^b^Adjusted for age, sex, BMI, smoking status, smoking intensity, stress, and all the food intakes

^c^Adjusted for age, sex, BMI, smoking status, smoking intensity, stress, intended physical exercise, and all the food intakes, except fat

^d^Adjusted for age, sex, BMI, smoking status, smoking intensity, stress, intended physical exercise, and all the food intakes, except carbohydrate

## Discussion

This study identified the risk factors for MetS based on six years of follow-up in a cohort of individuals who were not affected by the condition at baseline. By promoting BMI as a key risk factor for metabolic syndrome, policymakers can raise public awareness about the significance of maintaining a healthy weight, adopting healthier lifestyle and promoting physical activity levels. The intervention programs should be tailored to suit the unique needs of the Indonesian population, considering cultural and socioeconomic factors. Collaborative efforts between government agencies, healthcare providers, nutritionists, community organizations, and public health experts can ensure comprehensive and effective interventions that address the underlying causes of high BMI. Empowering primary care physicians with the necessary knowledge and resources to address BMI-related health issues can significantly reduce the burden of metabolic syndrome.

In this population-based cohort study, we investigated the association between risk factors and the survival rate for MetS, defined as the probability of living free from MetS. We demonstrated several principal findings, which are summarised as follows: during the 6-year follow-up period (1) the risk of developing MetS rose with increasing BMI. Obesity reduced the probability of living free from MetS by more than 50% and led to a 4.4-fold increased risk of developing MetS, (2) a sedentary lifestyle significantly increased MetS risk by 1.5 times and reduced the probability of living free from MetS by around 20%, (3) we observed that low and moderate fat intake had a protective effect against MetS and carbohydrate intake was a risk factor for developing MetS. However, this result needs to be carefully interpreted as the lower consumption of fat may be a consequence of having higher consumption of carbohydrate.

Previous studies have consistently reported higher BMI as predictor for MetS [37–39]. Other variables that have been reported in addition to BMI include sedentary lifestyle [40, 41], waist-to-hip ratio [42], older age, being female, and lower education level [39]. A population cohort in Korean adults suggested that dietary aspects, such as dietary diversity, was associated with the risk of MetS [43]. Diet and MetS association also supported by a cross-sectional study reported low fruit and vegetable intakes and high alcohol consumption as dietary factors that contribute to MetS risk, alongside high BMI and a sedentary lifestyle [40]. The findings of the present study are, in general, aligned with previous findings and suggested theories. While overweight-obesity and sedentary lifestyle were consistently reported in another population study, this study’s findings in relation to carbohydrate and fat intake as predictors of the future risk of MetS have not been widely reported in other studies. Different dietary patterns and types of carbohydrate and fat consumed in various populations allow diverse outcomes in the risk of MetS [40].

Previous observations related to fat intake and MetS were inconclusive. While some study proposed that high dietary fat intakes are associated with higher risk of MetS [44, 45], some other studies suggested the opposite [46, 47]. Based on a systematic review, the difference was due to the type of fat consumed, as higher polyunsaturated fatty acids (PUFA) was reported to be inversely associated with adiposity and obesity, as well as lower triglyceride levels, increased HDL, and an overall lower risk of MetS [45]. The current analysis shows that low and moderate fat intake lowers the risk of developing MetS. However, this result needs to be carefully interpreted as it could be depending to the type of fat (fatty acids) consumed, which this study did not measure, or considering the overall energy intake proportion, the protective effect of fat may be a consequence of having higher consumption of carbohydrate.

A dose–response meta-analysis of observational studies suggested that carbohydrate intake had a linear association with MetS risk, with a 2.6% increase in the risk of MetS per 5% energy from carbohydrate intake [48]. In addition, carbohydrate intake from starchy foods with a high glycaemic index (GI > 65) has been shown to contribute more strongly to metabolic disorders and hyperlipidaemia [49]. High consumption of carbohydrates has been consistently associated with a reduced HDL cholesterol level and increased plasma triglyceride levels [50, 51] mainly due to a higher triglyceride content in very low-density lipoprotein (VLDL) particles and overproduction of VLDL particles [52]. In addition to triglyceride and HDL levels, abdominal obesity is also strongly associated with carbohydrate intake [51]. This condition was likely relevant to participants in this study, who were, due to the prevalence of overweight and obesity, already at greater risk of abdominal obesity.

The abdominal obesity component was the most prevalent feature of MetS in this population and another reported MetS population [53]. It is representative of visceral adiposity which is a known risk factor for cardiovascular diseases. Although BMI does not represent adiposity, in the general population, overweight and obese BMI were correlated with increased levels of body fat [54]. Therefore, an individual with overweight-obesity was already at higher risk of abdominal obesity. Furthermore, increased adiposity has been theoretically linked to increased inflammation and metabolism suppression. The biochemical cascade was linked to glucose intolerance and hyperinsulinemia, as well as endothelial dysfunction, which is the predisposing factor for dyslipidaemia and vascular diseases [55, 56].

The present study showed that inadequate exercise had unfavourable effects on MetS risk. Although on the basis of their self-reported data, most participants had sufficient physical activity levels due to their employment as household assistants, when we clustered activity based on intentional physical exercise (playing sports or recreational exercise rather than activity associated with their employment), most participants did not meet recommended levels. Increased sedentary behaviour is associated with an increased risk of high levels of triglycerides, HDL-cholesterol, and fasting glucose [57, 58]. Conversely, around 30 min of moderate-intensity daily exercise improves factors related to MetS [59]. Men who have more than 4 times per week of vigorous, moderate, and strength exercise and more than 6 times per week of walking had a triglyceride level lower than 150 mg/dL [60, 61]. Furthermore, diastolic blood pressure [62] and fasting blood glucose [60] were reported to improve as a result of moderate exercise, particularly aerobic exercise [62].

In observing the development of MetS, we worked on several variables to avoid biases in measurement and analysis. We excluded participants who had limited or incomplete data on blood pressure, fasting blood glucose, HDL, and triglycerides and who were taking antihypertensive, antidiabetic, and cholesterol-lowering medications. We also excluded participants who did not fast prior to blood glucose testing. By doing this, we expected to better understand the incidence of MetS during six years of follow-up with observations every two years. However, we tried to involve as many relevant variables as possible to obtain a more accurate model.

The use of secondary data and limited power were among the limitations of this study. There are some potential sources of bias which lead to inaccuracies in the final results due to the self-reporting of some information, human error in data input, memory limitations, social desirability, intentional misreporting, or may not represent habitual diet, especially in the single 24-hour dietary recall. To manage with the data issue, we used a cleaning procedure to exclude missing information from the MetS criteria, such as instances, when a number was either too small or too excessive, as was the case with blood glucose levels, waist circumference, lipid profiles, and blood pressure, the smaller sample sizes were a result of the stringent data inclusion criteria to minimize follow-up loss and reduce bias. Therefore, the power was potentially affected (considered underpowered) by a smaller sample size, resulting from our rigorous participant selection process, even though the significances between the variables can be clearly produced. Other limitation was the cohort participation relies on the willingness to underwent routine medical checkups, which in population with low health-seeking behaviour such as in most of Indonesian community [63], may lead to bias in participant selection. Although with the large number of participants, and adjustment for potential confounding factors, including dietary intake and physical activity, the risk of selection bias was reduced. Another strength was that the anthropometric and clinical measurements were recorded by trained operators and are more accurate than self-reported measurements. In addition to that, the prospective cohort design employed in this study provided robust and longitudinal insights into the progression of MetS. After weighted adjustment, this study is a sound reflection of the epidemic characteristics of MetS among residents in Bogor District, Indonesia.

## Conclusions

Between 2011 and 2018, more than a quarter of the sample developed MetS. Overweight and obesity, insufficient exercise, and a higher quartile of carbohydrate consumption were observed as factors increasing the risk of MetS, even after controlling for several confounding factors. The highest cumulative survival of MetS was reported for participants with underweight BMI and the lowest cumulative survival was reported for participants with obese BMI. Given the importance of these findings, immediate strategic actions are needed to improve the existing early detection and monitoring programmes of NCD that promote community-based healthy lifestyle initiatives and is run by both the government and non-government organisations in Bogor District, Indonesia.

### Electronic supplementary material

Below is the link to the electronic supplementary material.

**Supplementary Material 1**. The 6-year Survival of MetS based on the Risk Factors

## Data Availability

The data that support the findings of this study are available from The Ministry of Health, Republic of Indonesia, under the Basic Health Research data, but restrictions apply to the availability of these data, which were used under license for the current study, and so are not publicly available. Data are however available with prior officially written permission of the Data Management Laboratory of NIHRD, Ministry of Health, Republic of Indonesia (datin.bkpk@kemkes.go.id).

## Abbreviations

BMI: Body Mass Index
HDL: High-Density Lipoprotein
MET: Metabolic Equivalent of Task
MetS: Metabolic Syndrome
MUFA: Monounsaturated Fatty Acid
NCEP ATP: National Cholesterol Education Program’s Adult Treatment Panel
NIHRD: National Institute of Health Research and Development
NCDs: Non-Communicable Diseases
PUFA: Polyunsaturated Fatty Acid
SFA: Saturated Fatty Acid
SDGs: Sustainable Development Goals
VLDL: Very Low-Density Lipoprotein
