# Supplementary material for: Body mass index as a dominant risk factor for metabolic syndrome among indonesian adults: a 6-year prospective cohort study of non-communicable diseases
Source: BMC Nutr. 2024 Mar 4;10:43. doi: 10.1186/s40795-024-00856-8 (PMC10913611; doi:10.1186/s40795-024-00856-8)
Supplement: Supplementary file 1 — Supplementary Material 1. The 6-year Survival of MetS based on the Risk Factors [file 40795_2024_856_MOESM1_ESM.docx]

Supplementary Material 1. The 6-year Survival of MetS based on the Risk Factors

| Risk Factors | Year | Risk Population | Cumulative Survival  (%) | 95% CI |
| --- | --- | --- | --- | --- |
| BMI |  |  |  |  |
| Normal | 2 | 500 | 91.7 | 89.1 – 93.8 |
|  | 4 | 491 | 90.1 | 87.3 – 92.3 |
|  | 6 | 470 | 86.2 | 83.1 – 88.9 |
| Underweight | 2 | 111 | 95.7 | 90.0 – 98.2 |
|  | 4 | 111 | 95.7 | 87.8 – 97.1 |
|  | 6 | 109 | 94.0 | 87.8 – 97.1 |
| Overweight | 2 | 217 | 84.4 | 79.4 – 88.3 |
|  | 4 | 208 | 80.9 | 75.6 – 85.2 |
|  | 6 | 177 | 68.9 | 62.8 – 74.1 |
| Obese | 2 | 304 | 66.4 | 61.9 – 70.5 |
|  | 4 | 274 | 59.8 | 55.2 – 64.2 |
|  | 6 | 226 | 49.3 | 44.7 – 53.8 |
| Age |  |  |  |  |
| < 50 | 2 | 880 | 81.5 | 79.0 – 83.7 |
|  | 4 | 843 | 78.1 | 75.5 – 80.4 |
|  | 6 | 761 | 70.5 | 67.6 – 73.1 |
| ≥ 50 | 2 | 252 | 85.1 | 80.6 – 88.7 |
|  | 4 | 241 | 81.4 | 76.5 – 85.4 |
|  | 6 | 221 | 74.7 | 69.3 – 79.2 |
| Sex |  |  |  |  |
| Male | 2 | 406 | 87.5 | 84.1 – 90.2 |
|  | 4 | 394 | 84.9 | 81.3 – 87.9 |
|  | 6 | 359 | 77.4 | 73.3 – 80.9 |
| Female | 2 | 726 | 79.6 | 76.8 – 82.1 |
|  | 4 | 690 | 75.7 | 72.7 – 78.3 |
|  | 6 | 623 | 68.3 | 65.2 – 71.2 |
| Tobacco habit |  |  |  |  |
| Never smoke | 2 | 542 | 80.2 | 77.0 – 83.0 |
|  | 4 | 514 | 76.0 | 72.6 – 79.1 |
|  | 6 | 458 | 67.8 | 64.1 – 71.1 |
| Ever smoke | 2 | 190 | 83.7 | 78.2 – 87.9 |
|  | 4 | 179 | 78.9 | 73.0 – 80.5 |
|  | 6 | 160 | 70.5 | 64.1 – 76.0 |
| Smoke | 2 | 400 | 84.6 | 81.0 – 87.5 |
|  | 4 | 391 | 82.7 | 79.0 – 85.8 |
|  | 6 | 364 | 77.0 | 72.9 – 80.5 |
| Smoking Intensity |  |  |  |  |
| Not smoking | 2 | 778 | 81.0 | 78.4 – 83.4 |
|  | 4 | 739 | 77.0 | 74.2 – 79.5 |
|  | 6 | 660 | 68.8 | 65.7 – 71.6 |
| 1-9 cigarette(s) per day | 2 | 191 | 84.5 | 79.1 – 88.6 |
|  | 4 | 187 | 82.7 | 77.2 – 87.1 |
|  | 6 | 174 | 77.0 | 70.9 – 82.0 |
| 10-19 cigarettes per day | 2 | 136 | 85.0 | 78.5 – 89.7 |
|  | 4 | 133 | 83.1 | 76.4 – 88.1 |
|  | 6 | 125 | 78.1 | 70.9 – 83.8 |
| ≥ 20 cigarettes per day | 2 | 27 | 90.0 | 72.1 – 96.7 |
|  | 4 | 25 | 83.3 | 64.5 – 92.7 |
|  | 6 | 23 | 76.7 | 57.2 – 88.1 |
| Stress |  |  |  |  |
| No | 2 | 661 | 82.8 | 80.0 – 85.3 |
|  | 4 | 631 | 79.1 | 76.1 – 81.7 |
|  | 6 | 572 | 71.7 | 68.4 – 74.7 |
| Yes | 2 | 471 | 81.5 | 78.1 – 84.4 |
|  | 4 | 453 | 78.4 | 74.8 – 81.5 |
|  | 6 | 410 | 70.9 | 67.1 – 74.5 |
| Physical Activity |  |  |  |  |
| Enough | 2 | 1017 | 81.6 | 79.4 – 83.7 |
|  | 4 | 970 | 77.9 | 75.4 – 80.1 |
|  | 6 | 877 | 70.4 | 67.8 – 72.8 |
| Not enough | 2 | 115 | 88.5 | 81.6 – 92.9 |
|  | 4 | 114 | 87.7 | 80.7 – 92.3 |
|  | 6 | 105 | 80.7 | 72.9 – 86.6 |
| Intended Physical Exercise |  |  |  |  |
| Enough | 2 | 137 | 86.7 | 80.4 – 91.1 |
|  | 4 | 131 | 82.9 | 76.1 – 88.0 |
|  | 6 | 124 | 78.5 | 71.2 – 84.1 |
| Not enough | 2 | 995 | 81.7 | 79.4 – 83.7 |
|  | 4 | 953 | 78.2 | 75.8 – 80.5 |
|  | 6 | 858 | 70.4 | 67.8 – 72.9 |
| Food Intake |  |  |  |  |
| Energy |  |  |  |  |
| Quartile-1 | 2 | 275 | 79.9 | 75.3 – 83.8 |
|  | 4 | 268 | 77.9 | 73.1 – 81.9 |
|  | 6 | 246 | 71.5 | 66.4 – 76.0 |
| Quartile-2 | 2 | 288 | 83.7 | 79.4 – 87.2 |
|  | 4 | 277 | 80.5 | 75.9 – 84.3 |
|  | 6 | 253 | 73.6 | 68.6 – 77.9 |
| Quartile-3 | 2 | 284 | 82.6 | 78.1 – 86.2 |
|  | 4 | 267 | 77.6 | 72.8 – 81.7 |
|  | 6 | 245 | 71.2 | 66.1 – 75.7 |
| Quartile-4 | 2 | 285 | 82.9 | 78.4 – 86.4 |
|  | 4 | 272 | 79.1 | 74.4 – 83.0 |
|  | 6 | 238 | 69.2 | 64.0 – 73.8 |
| Protein |  |  |  |  |
| Quartile-1 | 2 | 283 | 82.3 | 77.8 – 85.9 |
|  | 4 | 274 | 79.7 | 75.0 – 83.5 |
|  | 6 | 255 | 74.1 | 69.2 – 78.4 |
| Quartile-2 | 2 | 279 | 81.1 | 73.5 – 82.2 |
|  | 4 | 269 | 78.2 | 67.0 – 76.5 |
|  | 6 | 248 | 72.1 | 77.8 – 85.9 |
| Quartile-3 | 2 | 283 | 82.3 | 73.1 – 81.9 |
|  | 4 | 268 | 77.9 | 64.3 – 74.1 |
|  | 6 | 239 | 69.5 | 79.1 – 87.0 |
| Quartile-4 | 2 | 287 | 83.4 | 74.7 – 83.3 |
|  | 4 | 273 | 79.4 | 64.6 – 74.3 |
|  | 6 | 240 | 69.8 |  |
| Fat |  |  |  |  |
| Quartile-1 | 2 | 273 | 79.4 | 74.7 – 83.3 |
|  | 4 | 266 | 77.3 | 72.5 – 81.4 |
|  | 6 | 243 | 70.6 | 65.5 – 75.2 |
| Quartile-2 | 2 | 293 | 85.2 | 81.0 – 88.5 |
|  | 4 | 283 | 82.3 | 77.8 – 85.9 |
|  | 6 | 261 | 75.9 | 71.0 – 80.1 |
| Quartile-3 | 2 | 281 | 81.7 | 77.2 – 85.4 |
|  | 4 | 262 | 76.02 | 71.3 – 80.3 |
|  | 6 | 236 | 68.6 | 63.4 – 73.2 |
| Quartile-4 | 2 | 285 | 82.9 | 78.4 – 86.4 |
|  | 4 | 273 | 79.4 | 74.7 – 83.3 |
|  | 6 | 242 | 70.4 | 65.2 – 74.9 |
| Carbohydrate |  |  |  |  |
| Quartile-1 | 2 | 285 | 82.9 | 78.4 – 86.4 |
|  | 4 | 277 | 80.5 | 75.9 – 84.3 |
|  | 6 | 253 | 73.6 | 68.6 – 77.9 |
| Quartile-2 | 2 | 276 | 80.2 | 75.6 – 84.1 |
|  | 4 | 265 | 77.0 | 72.2 – 81.1 |
|  | 6 | 244 | 70.9 | 65.8 – 75.4 |
| Quartile-3 | 2 | 287 | 83.0 | 79.1 – 87.0 |
|  | 4 | 271 | 78.8 | 74.1 – 82.7 |
|  | 6 | 246 | 71.5 | 66.4 – 76.0 |
| Quartile-4 | 2 | 284 | 82.6 | 78.1 – 86.2 |
|  | 4 | 271 | 78.8 | 74.1 – 82.7 |
|  | 6 | 239 | 69.5 | 64.3 – 74.1 |
| Sodium |  |  |  |  |
| Quartile-1 | 2 | 295 | 85.8 | 81.6 – 89.0 |
|  | 4 | 282 | 82.0 | 77.5 – 85.7 |
|  | 6 | 257 | 74.7 | 69.8 – 79.0 |
| Quartile-2 | 2 | 282 | 82.0 | 77.5 – 85.7 |
|  | 4 | 271 | 78.8 | 74.1 – 82.7 |
|  | 6 | 248 | 72.1 | 67.0 – 76.5 |
| Quartile-3 | 2 | 283 | 82.3 | 77.8 – 85.9 |
|  | 4 | 273 | 79.4 | 74.7 – 83.3 |
|  | 6 | 243 | 70.6 | 65.5 – 75.2 |
| Quartile-4 | 2 | 272 | 79.1 | 74.4 – 83.0 |
|  | 4 | 258 | 75.0 | 70.1 – 79.2 |
|  | 6 | 234 | 68.0 | 62.8 – 72.7 |
